# Policy synergy and thematic evolution of strategic mineral policies in China

**DOI:** 10.1016/j.fmre.2024.11.017

**Published:** 2024-12-25

**Authors:** Xiaolei Sun, Yingjie Sheng, Guoqiang Li, Qiang Ji

**Affiliations:** a Institutes of Science and Development, Chinese Academy of Sciences, Beijing 100190, China; b School of Public Policy and Management, University of Chinese Academy of Sciences, Beijing 100049, China; c National Science Library, Chinese Academy of Sciences, Beijing 100190, China

**Keywords:** Strategic critical minerals, Policy analysis, Policy synergy, Policy attention, Network analysis, Text mining

## Abstract

The significance of strategic critical minerals in the global transition toward green and low-carbon energy has attracted considerable attention from many countries. Understanding the evolution of the strategic critical minerals policy system is crucial for identifying key issues in policymaking. This study examines 984 Chinese policy texts from 2011 to 2023 within the framework of “policy theme-policy synergy” and elucidates the evolutionary trajectory of China's strategic critical mineral policy system. The results indicate that China's strategic critical minerals policy has consistently centered around technology and enterprises in terms of policy themes, with a growing emphasis on green and low-carbon initiatives. Policies mainly focus on coal, oil, natural gas, and rare earth, while paying less attention to the significance of strategic critical minerals in energy transition. China has established a tighter policy synergy network for jointly formulating strategic critical minerals. However, as a key government department, the Ministry of Natural Resources has yet to demonstrate its central position and requires stronger synergies with other sectors. These findings provide a decision-making reference for China's strategic minerals policy development, design, and optimization.

## Graphical abstract

**Figure:**
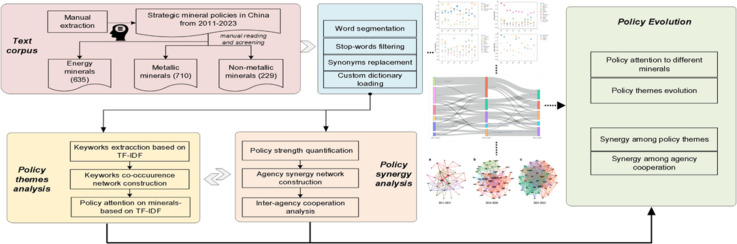

## 1 Introduction

In response to climate change, the transition toward green energy has emerged as a crucial strategic initiative for most nations [1,2], driven by the reliance on various critical minerals [3]. Consequently, there has been an exponential surge in demand for these minerals [4,5]. Furthermore, due to their uneven distribution and the resulting supply-demand imbalance, there are new challenges to the security of global mineral resource supplies [6]. As international competition for these resources intensifies [7], major economies are implementing new policies to diversify their mineral supplies [8]. These policies involve evaluating the criticality of mineral products and key raw materials [9], continuously updating critical mineral lists, and formulating long-term plans for the development, utilization, and strategic reserves of these vital resources [[10], [11], [12]].

Ensuring the accuracy and effectiveness of strategy and policy formulation is critical for securing strategic critical mineral resources. Therefore, by conducting a comprehensive study of China's strategic critical mineral policy system, we aim to deepen our understanding of policy formulation, reassess current policy frameworks, extract valuable insights for the advancement of the strategic critical mineral sector, and equip policymakers with the necessary knowledge for informed decision-making [13,14].

Research on China's strategic critical minerals policy can be categorized into three primary areas. The first type of research examines the content of policies related to specific minerals or sectors associated with critical minerals [15,16]. The second type focuses on the policy development process [17]. The third explores the historical evolution of policies in particular minerals or related fields, such as rare earth policies [18,19], urban minerals policies [20], and policies relating to mining's role in the circular economy [21].

However, most of the existing research focuses on specific policy content or causes related to particular mineral sectors, with limited attention given to the evolutionary dynamics of China's strategic critical mineral policy system from a synergistic perspective. Little emphasis has been placed on examining interagency synergy during policy formulation and implementation, hindering our understanding of changes within the policy system. The evolution of the policy system is a formalized process in which government agencies generate new regulations. These newly established regulations facilitate interagency cooperation and further shape policy implementation [22]. Policy synergy is a model for solving cross-sectoral problems based on the theory of synergy, which is the process of coordinating policies and actions among different actors and government agencies to achieve common goals [[23], [24], [25]]. China's policymaking system consists of multiple government agencies at different levels, and the agencies involved reflect the breadth and strength of policy influence. Moreover, due to the extensive industry chain and diverse fields involved in strategic critical minerals, the complexity and multidimensional nature of its policy system renders it challenging for a single government department to effectively support and promote the development of the entire industry. Enhancing synergy among departments responsible for strategic critical mineral policymaking presents a challenge for both government agencies and academia. This synergy is crucial for the successful implementation of policies and the safeguarding of national strategic mineral resources. Therefore, exploring the cooperation and synergy between different government agencies from the perspective of policy synergy can help us to understand the evolution process of the policy system and analyze how the synergy between departments supports the development of the policy system [26].

In terms of research methods, most studies on China's critical mineral policies primarily utilize qualitative research methodologies such as content analysis. Scholars interpret policy texts through manual coding and other systematic approaches [21]. However, manual analysis poses challenges when dealing with a substantial volume of policy texts encompassing multiple fields, stakeholders, and an extended period. This approach fails to facilitate subsequent policy document interpretation [14] or enable the exploration of interagency relationships within the policy network [27].

In contrast, the application of quantitative analysis to policy documents facilitates the discovery of the underlying factors driving the overall political process and enables the tracking of policy evolution [28]. Social network analysis is particularly effective for mapping the actors and their interrelationships within a network, facilitating the identification of distinct actors and small groups that hold significance in the network. This analytical approach contributes to a better understanding of the intricate evolution of policy networks [29]. In addition, co-word and network analysis methods help identify associations between topics within policy documents, enabling the exploration of the evolving patterns of policy themes [30].

Many scholars have started applying these quantitative methods to other areas of policy research with promising results. Huang et al. [31] conducted co-occurrence and cluster analyses to examine the evolution of China's science, technology, and innovation policy in terms of its thematic and focal areas. Huang et al. [32] utilized social network analysis to construct a “policy target-policy instrument” network for China's nuclear energy policy, which identified the core policy targets and instruments at various stages. Liu and Wang [23] employed text mining and social network analysis methods to examine the degree of synergy concerning China's policies on coal de-capacity. The above studies provide novel technical tools for this study to investigate the synergistic and thematic evolution of strategic and critical mineral policies.

To conclude, considering the inadequate attention given to policy synergy and the absence of quantitative analysis in existing research on China's strategic critical mineral policies, this study introduces text mining and social network analysis as quantitative methods of policy analysis. The objective is to explore policy evolution through the lens of “policy theme-policy synergy.” Specifically, this study addresses two key research questions: (1) What are the thematic focuses and priorities of China's strategic critical minerals policies at different periods? (2) How is China's policymaking system for strategic critical minerals evolving?

This study contributes to the existing literature in three ways: (1) Unlike previous studies that focus primarily on the policy content of specific mineral fields, this study establishes a comprehensive policy text database including China's strategic critical mineral policies from 2011 to June 2023, which correspond to the 24 strategic critical minerals outlined in the National Mineral Resources Plan for 2016–2020. (2) It introduces the perspective of policy synergy, developing an analytical framework for “policy theme-policy coordination,” which identifies the stage characteristics of China's strategic critical mineral policy network and focuses on the coordination relationship among core government agencies. This framework aims to provide valuable insights for industry-wide systematic planning and collaborative governance in the strategic critical minerals sector. (3) In contrast to previous studies that primarily rely on qualitative content analysis, this research employs an integrated approach combining text mining and social network analysis techniques. By constructing co-word networks and sectoral synergy networks based on 984 policy texts related to energy and both metallic and nonmetallic minerals, this study provides objective evidence for identifying policy themes, core government agencies, and their evolution over time.

The subsequent sections of this study are organized as follows. The second section presents the research design and data utilized in this study. The third section provides an overview of the analysis results and discussion. Finally, the fourth section summarizes the findings of this research and proposes corresponding strategies and recommendations.

## 2 Research design and data

**Fig. 1:**
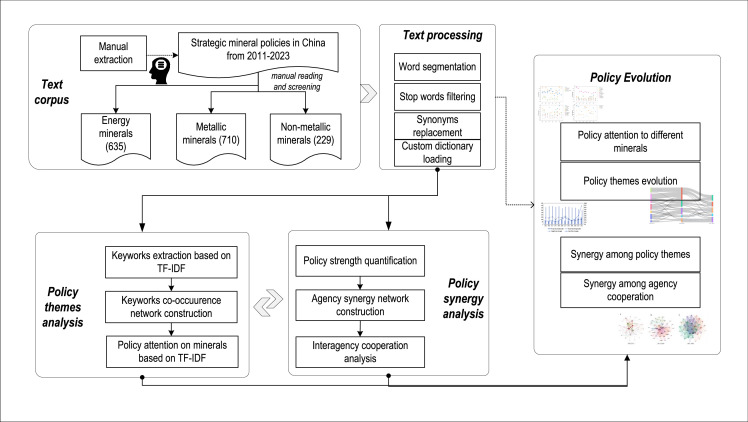
**Research Framework**.

In this study, we develop a “policy theme-policy synergy” framework to explore the evolution of China's strategic critical mineral policies, as outlined in the four key steps shown in Fig. 1. First, a textual corpus was constructed by collecting policy texts related to strategic critical minerals from the PKULAW database, with final selections made through manual review. Second, text processing was conducted, including word segmentation, stop words filtering, synonym replacement, and loading a custom dictionary. Third, both policy themes and policy synergy were analyzed. From a thematic perspective, the importance of policy themes was captured based on policy keywords and keyword co-occurrence networks; the level of policy attention given to different minerals was assessed using the TF-IDF algorithm. From the synergy perspective, policy strength was quantified, and a policy agency synergy network was created to analyze the structure of interagency cooperation. Finally, the evolution of policy themes and synergies was visualized, providing insights into the policy development trajectory.

### 2.1 Policy themes

Identifying the core themes in the policy documents [14] is essential for fully understanding the content, conditions, and historical context of policies outlined in these documents. This identification not only aids in recognizing current weaknesses but also provides a foundation for future adjustment [33]. The policy themes represent the essential content of the document, highlighting its focus and direction [34]. Since policy texts often address multiple themes, and a single theme may recur across various policy documents, this study further breaks down and analyzes policy themes through two components: theme clustering and policy attention. Theme clustering involves extracting and categorizing complex policy texts into distinct themes [35], facilitating in-depth research. Policy attention, on the other hand, refers to the emphasis placed on specific minerals in strategic critical minerals policies. Thus, this study explores the evolution of strategic critical mineral policies over different stages by examining both the clustering of policy themes and the level of policy attention dedicated to individual minerals.

This study employs text mining techniques to extract keywords from policy documents across different stages. It constructs co-occurrence networks of these keywords and computes the level of policy attention given to each mineral to analyze the development of policy themes. The analysis is conducted in five key steps:

Step 1: Text processing. The policy text is processed using the “jieba” tool, which includes word segmentation, stop-words filtering, synonym replacement, and custom dictionary loading.

Step 2: Keyword extraction. Keywords for each policy document are extracted using the term frequency-inverse document frequency (TF-IDF) algorithm. Specifically, the top eight keywords with the highest TF-IDF values are selected to construct the keyword co-occurrence network. The TF-IDF algorithm is a weighted statistical method used in information retrieval and text mining to evaluate the significance of a word within a document set or corpus. The significance of a word increases proportionally with its frequency within the document set but decreases inversely to the frequency of documents in which it occurs within the corpus [36]. It is calculated using the following formula:

(1) $$\begin{array}{c} \mathrm{tfid}f_{i,j}=tf_{i,j}\times \mathrm{id}f_{i,j} \end{array}$$

where $tf_{i,j}$ is the term frequency of feature word $t_{i}$ occurring in text $d_{j}$, calculated as:

(2) $$tf_{i,j}=\frac{n_{i,j}}{\sum_{k}n_{k,j}}\;$$

where $n_{i,j}$ denotes the frequency of feature word $t_{i}$ occurring in text $d_{j}$, and $\sum_{k}n_{k,j}$ is the total number of feature words in text $d_{j}$.

The inverse document frequency $idf_{i,j}$ is the logarithm of the total number of documents in the corpus divided by the number of documents containing the term, computed as:

(3) $$idf_{i,j}=\log\frac{\left|D\right|}{1+\left|j:t_{i\;}\in d_{j}\right|}$$

where |D| represents the total number of documents in the corpus, and $|j:t_{i\;}\in d_{j}|$ represents the number of documents containing the feature word $t_{i}$.

Step 3: Keyword co-occurrence network construction. Keyword co-occurrence features are computed for different time periods. In these networks, each node represents a policy keyword, while each edge represents the co-occurrence relationship between two policy keywords. By examining the co-occurrence relationship, the primary policy themes at each stage can be identified, illustrating the evolution of policy themes over time.

Step 4: Centrality indicator computation and evolution analysis. The eigenvector centrality of each node in the co-occurrence network is calculated to determine the most influential keywords. Eigenvector centrality measures the importance of a node based on both the number of connections it has and the importance of the nodes to which it is connected [37]. It is calculated as shown in Eq. (4):

(4) $$E_{i}=\;\frac{1}{\lambda }\sum_{j\epsilon N\left(i\right)}a_{ij}E_{j}$$

where $E_{i}$ is the eigenvector centrality of keyword i, A is the adjacency matrix of the keyword co-occurrence network, and $a_{ij}$ is the element in the adjacency matrix A. If there is a co-occurrence relationship between i and j, then $a_{ij}>0$, otherwise $a_{ij}=0$. λ is the eigenvalue, and N(i) is the set of other keywords directly connected to keyword i. Keywords with higher eigenvector centrality indicate higher significance within the network. In this study, we utilize eigenvector centrality to indicate the size of node labels in the keyword co-occurrence network, where larger node labels signify greater significance of the keyword's position within the network.

Step 5: Policy attention analysis. The TF-IDF algorithm is applied to calculate the TF-IDF values of 24 strategically critical minerals. These values reflect the relative importance of each mineral within the policy text, providing insights into the level of attention devoted to each mineral over time.

### 2.2 Policy synergy

This study adopts an interagency synergy perspective to analyze the evolution of China's policy system for strategic critical minerals, focusing on how collaboration among government agencies has contributed to policymaking processes.

The following steps outline the approach to analyzing the policy synergy:

Step 1: Quantifying policy strength. This study examines the synergy among government agencies in formulating China's strategic critical minerals policy. Policy synergy refers to cases where multiple agencies jointly issue a policy. To quantitatively analyze the policy agency synergy, we employ the policy strength indicator $P_{j}$, which measures the efficacy of policy documents. Policy strength reflects the legal and implementation efficacy of a policy. The higher the level of the policymaking agency, the greater its authority and policy influence are generally believed to be, resulting in policies formulated by it receiving more attention from government agencies. Policies issued jointly by multiple government agencies often signal complex, cross-sectoral issues requiring coordination across departments [38]. Thus, the effectiveness of the policy is intricately tied to the cross-sectoral synergy within the government. To quantify policy strength, we apply the indicator division method proposed by Peng et al. [39]. Table 1 illustrates the indicator system, which categorizes policy strength into five levels based on the type of policy document. Each policy document is scored individually by the level of the department responsible for its formulation.

**Table 1:** **Policy strength quantitative indicator system**.

Type of policy	Indicator score
Legal documents issued by the National People's Congress	5
Plans, opinions, approaches, and programs issued by the State Council	4
Plans, opinions, approaches, and programs issued by ministries and commissions	3
Guidelines, norms, and standards issued by ministries and commissions, as well as approvals, decisions, and reports issued by the State Council	2
Notifications, reports, letters, and replies issued by ministries and commissions	1

This study classifies policy issuance structures into two types: single-agency and joint issuance. It introduces a joint issuance policy strength index to assess the overall strength of policies at different stages, capturing changes in departmental synergy during policy formulation over time. A greater intensity of joint policy issuance signifies a stronger degree of synergy among government agencies. This synergy is quantified using the following equation:

(5) $$\begin{array}{c} PS_{i}=\;\sum_{j=1}^{N}B_{\mathrm{ji}}\times P_{\mathrm{ji}},i\in \left[2011,2023\right] \end{array}$$

where $PS_{i}$ represents the collective strength of policies issued jointly in year i, N is the number of such polices, $B_{ji}$ is the number of agencies jointly issuing policies in item j, and $P_{ji}$ represents the strength of policies issued jointly in item j.

To analyze the core policymaking agencies that formulate strategic critical minerals policy, this study employs social network analysis to construct a joint policy network. Our approach involves the following steps:

Step 2: Constructing the government agencies synergy network and analyzing network metrics. This study constructs a policy synergy network of strategic critical minerals at various stages using Gephi software, where each node represents a policy making agency and the edges represent the joint relationships between agencies. The thickness of the edges indicates the frequency of joint policies issued and the proximity of agencies’ relationships.

We analyzed the evolution of synergy network through density, average distance, and average clustering coefficient indicators. These indicators are calculated as shown in Eqs. (6) – (9), respectively:

(6) $$Density=\frac{2E}{N\left(N-1\right)}$$

where Density is the network density, E is the total number of edges in the network, and N is the total number of nodes. In the joint policy network, density reflects the overall tightness of collaboration among policy agencies.

(7) $$APL=\;\frac{1}{N\left(N-1\right)}\sum_{i\neq j}d\left(i,j\right)$$

where APL is the average distance of the network, and d(i,j) is the shortest path length between node i and node j. The average distance of the joint policy network reflects the efficiency of information or influence dissemination among policy agencies. The smaller the average distance is, the more closely the nodes in the network are connected to each other and the more efficiently information or resources are transferred.

(8) $$C=\;\frac{1}{m}\sum_{i=1}^{n}\frac{1}{k_{i}\left(k_{i}-1\right)}$$

where C represents the average clustering coefficient of the entire network, nis the total number of nodes in the network, $k_{i}$ is the degree of node i, and mis the total number of edges in the network. The average clustering coefficient reflects the tendency of policy agencies to form collaborative groups. A higher average clustering coefficient indicates that policy agencies are more likely to form tight-knit collaborative groups, promoting policy consistency and synergy.

Step 3: Computing the betweenness centrality of each node in the joint policy network to identify the core, or the most influential node within the network. Betweenness centrality evaluates the significance of a node in a network by measuring its capacity to act as an intermediary [40], which is calculated as shown in Eq. 9:

(9) $$BC\left(v\right)=\;\sum_{s\neq v\neq t}\frac{\sigma_{\;st}\left(v\right)}{\sigma_{\;st}}$$

where BC(v) is the betweenness centrality of node v, s and t are nodes in the network other than node v, $\sigma_{\;st}$ is the number of shortest paths from node s to node t, and $\sigma_{\;st}\left(v\right)$ is the number of shortest paths from node s to node t that passes through node v.

In the joint policy network, agencies with higher betweenness centrality play a more crucial role in facilitating information transfer among other agencies. Therefore, betweenness centrality can serve as a tool to identify sectors that function as intermediaries among different sectors and possess the ability to harmonize the interests of all parties involved. In this paper, we use betweenness centrality as a measure of network node labels, where larger nodes labels indicate the greater importance of a policy agency within the network.

Step 4: Examining the evolution of core policy agencies within the network by comparing the top nodes in terms of betweenness centrality.

### 2.3 Data

The data utilized in this study are derived from policy documents pertaining to strategic critical minerals that have been issued by the Central Government of China, as well as departments under the State Council and its national bureaus. The National Mineral Resources Plan (2016–2020) [41] identifies 24 minerals—including oil, natural gas, coal, rare earths, and crystalline graphite—as strategic resources that are subject to macro-control, supervision, and mineral resource management. Based on this classification, this study defines strategic critical mineral policies as policy documents that address the deployment of arrangements for nationally listed strategic minerals.

The collection of policy texts includes the following steps. First, this study utilized the PKULAW database to identify policies related to strategic critical minerals issued by the Central Government of China, as well as its national bureaus and departments under the State Council, from January 1, 2011, to June 2023. The keywords used for policy titles were “mineral resources” and “mining,” while the full-text policies were searched using keywords such as “mineral resources,” “mining,” “energy resources,” “metals,” “nonmetallic,” “rare earths,” and “graphite.” A total of 1,435 policies were preliminarily identified.

Next, strategic critical mineral policies were categorized into energy mineral policies, metallic mineral policies, and nonmetallic minerals as categorized in the National Mineral Resources Plan (2016–2020) [41] and shown in Table 2. This study employed “energy minerals,” “metallic minerals,” and “nonmetallic minerals” as keywords along with the names of 24 specific minerals to conduct a comprehensive search across 1,435 policy texts. Specifically, if any keyword of a certain type of mineral appeared in the full text, we incorporated it into this type of mineral policy.

**Table 2:** **Critical Minerals Category in the National Mineral Resources Plan (2016–2020)**.

Type of minerals	Name of mineral species
Energy minerals	oil, natural gas, shale gas, coal, coal bed methane, uranium
Metallic minerals	iron, chromium, copper, aluminum, gold, nickel, tungsten, tin, molybdenum, antimony, cobalt, lithium, rare earths, zirconium
Nonmetallic minerals	phosphorus, potash, crystalline graphite, fluorite

Finally, we employed manual reading and screening methods to eliminate policy documents that exhibited ambiguous content or lacked high relevance to strategic critical minerals. A total of 984 policies related to energy minerals (635), metallic minerals (710), and nonmetallic minerals (229) were collected and screened for use as the research object in this paper.

**Fig. 2:**
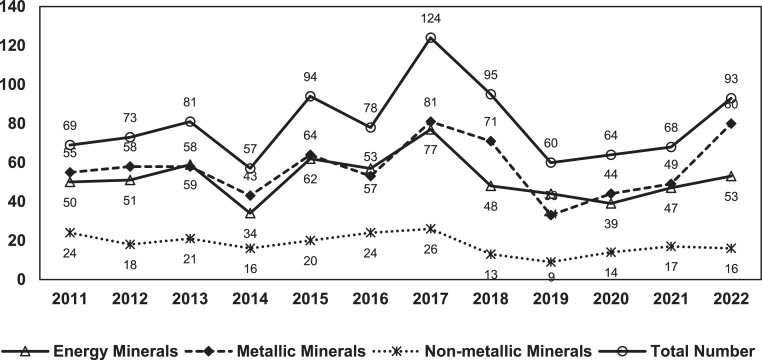
**Trends in strategic critical minerals policy releases, 2011–2022**.

Fig. 2 depicts the temporal evolution of China's policies on strategic critical minerals from 2011 to 2022. We have divided the timespan into three phases according to China's Five-Year Planning: the 12th Five-Year plan from 2011 to 2015, the 13th Five-Year Plan from 2016 to 2020, and the 14th Five-Year Plan from 2021 to 2023. As the policy text data were collected up until June 2023, we have excluded incomplete 2023 data from Fig. 2 to better reflect the trend in strategic critical mineral policies. As shown in Fig. 2, the quantity of China's policies on strategic critical minerals has exhibited a fluctuating trend. The year 2016 marked the beginning of the 13th Five-Year Plan period, during which several policies were implemented, including the National Mineral Plan (2016–2020). Afterwards, in response to the policy requirements of the central government, each province introduced its own master plan for mineral resource management. These plans received a reply from the Ministry of Resources and Land, resulting in a peak number of policies in 2017. The State Council carried out institutional reforms in 2018, and departments went through a series of processes such as consolidation of responsibilities and new construction. 2018 was a transitional period; therefore, the number of policy releases declined in relative terms. The number of policy releases rose again after 2019, returning to the normal levels before the institutional reforms.

## 3 Results and discussion

### 3.1 The evolution of policy themes

**Fig. 3:**
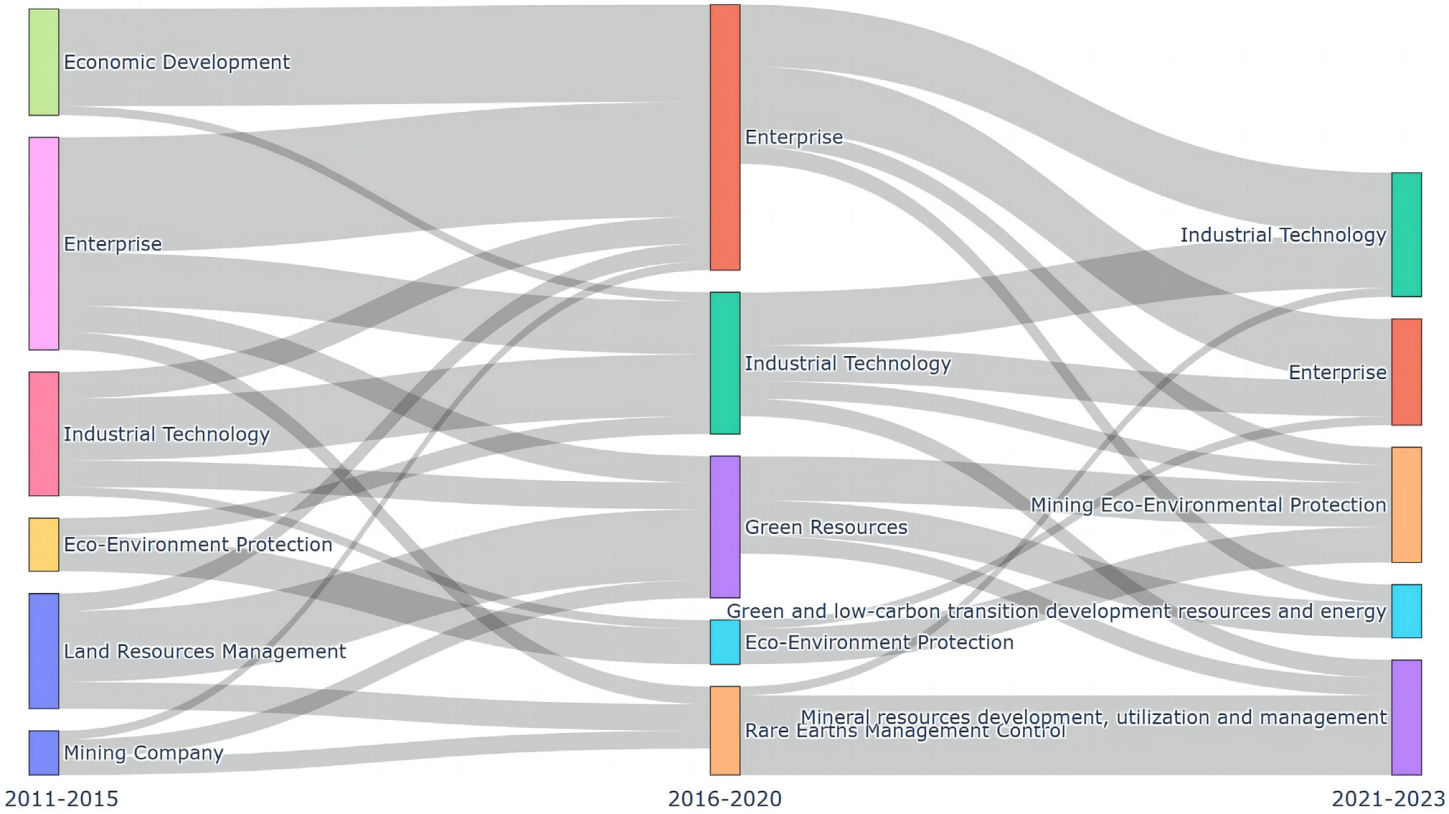
**Evolution of strategic critical minerals policy themes**. The width of the stream in the figure indicates the number of keywords that are transferred from one topic to another, and the nodes indicate the different stages of topics.

The extraction of keywords from policy texts and the visualization of keyword co-occurrence networks are primarily used to analyze the evolution of strategic critical minerals policy themes, with results shown in Fig. 3. The top 20 keywords in the co-occurrence network for each period from 2011 to 2023 are presented in Table 3. The nodes in Fig. 3 indicate policy themes at varying periods, while the width of the stream indicates the quantity of keywords transitioning from one theme to another.

**Table 3:** **Top 20 keywords in the keywork co-occurrence networks from 2011 to 2023**.

2011–2015		2016–2020		2021–2023	
Keyword	Eigenvector Centrality	Policy Keyword	Eigenvector Centrality	Policy Keyword	Eigenvector Centrality
Enterprise	0.558	Enterprise	0.505	Enterprise	0.430
Technology	0.342	Technology	0.337	Highlights	0.351
Industries	0.290	Highlights	0.282	Technology	0.348
Rare Earths	0.253	Industry	0.282	Systems	0.255
Products	0.241	Industries	0.259	National	0.251
Industry	0.215	Product	0.243	Green	0.244
Industrial	0.190	Materials	0.210	Utilization	0.235
Materials	0.183	Industrial	0.188	Industries	0.219
Ltd.	0.168	Resources	0.181	Industry	0.210
Situation	0.141	Standards	0.150	Industrial	0.187
Utilization	0.123	Rare Earths	0.129	Resources	0.141
Shares	0.120	Projects	0.119	Energy	0.139
Coal Mine	0.108	Areas	0.119	Policy	0.128
Declaration	0.106	Green	0.103	Economy	0.121
Environment	0.102	Utilization	0.100	Standards	0.119
Company	0.092	Ltd.	0.099	Projects	0.112
Unit	0.087	Region	0.096	Products	0.108
Economy	0.086	Departments	0.094	Eco	0.107
System	0.085	Systems	0.091	Green Low Carbon	0.096
Region	0.082	Equipment	0.088	Carbon Peaking	0.066

It can be seen that the evolution types of policy themes include continuation, differentiation, merger, and other trends, indicating that the policy focus of strategic critical minerals is developed on the basis of the continuous deepening of previous achievements. By comparing the evolution of policy themes and keywords at each stage of China's strategic critical minerals policies, we have observed that the past decade has witnessed diverse development needs and contextual conditions for China's strategic critical minerals, resulting in distinct focal points within the policy at different periods. It is noteworthy that “enterprise” and “technology” have consistently remained the most fundamental keywords in the policy text, highlighting the enduring emphasis on promoting and innovating enterprise and industrial technologies within the government.

From 2011 to 2015, in addition to the core themes of “enterprise” and “industrial technology,” policies also highlight the pivotal role of mining in propelling economic and social progress. Furthermore, the emergence of the “ecological environment protection” theme cluster signifies China's recognition during this stage of the ecological impact caused by mineral development and utilization. Consequently, a series of policies were implemented to address environmental protection, ecology, and pollution-related concerns. The mining, smelting, and production processes of minerals can result in a range of ecological and environmental issues. These include the release of pollutants such as wastewater, exhaust gas, and slag, which cause significant contamination to surface water, groundwater, atmosphere, and agricultural land [42]. As a significant producer and exporter of critical minerals with strategic importance, China has been facing substantial ecological and environmental pressures while also bearing the environmental costs associated with this production [43]. As a result, during the 12th Five-Year Plan, China introduced a series of policies, including the 12th Five-Year Plan for National Environmental Protection, which specifically emphasized the regulation of resource development in terms of ecological and environmental concerns.

From 2016 to 2020, the government increasingly emphasized the crucial role of enterprises in the mineral development process. Simultaneously, the theme of “economic development” has merged into “enterprise,” thereby highlighting their interdependent relationship. “Rare earths management control” emerged as a novel policy theme, reflecting the implementation of more targeted measures for rare earth resources governance. During the 13th Five-Year Plan period, China's regulatory focus on the rare earth industry shifted more from the export segment to domestic production. The competent authorities responded to this shift by implementing robust measures to enhance the management of rare earth resource extraction. Key aspects of these measures included closely monitoring the total mining volume, total production volume, control targets, and export licenses related to rare earths [44]. Furthermore, the emergence of the topic “green resources” suggests that the Chinese government has begun implementing green development practices, with a focus on promoting sustainable and environmentally friendly transformations within the resource industry.

During the period of 2021–2023, the theme of “mineral resources development, utilization, and management” emerged as a focal point, stemming from the convergence of “industrial technology,” “rare earths management control,” and “green resources.” This signifies that the Chinese government has taken into consideration sustainable industrial technology development and green resource practices, while emphasizing efficient resource utilization in mineral resource development, utilization, and management. The theme of “green and low-carbon transformation and development of resources and energy”—with “green,” “resources,” “utilization,” “energy,” and “peak carbon” as core keywords—has become the new focus of strategic and critical mineral policies during this period. The issuance of the carbon peak action plan before 2030 was announced by the State Council, which includes the implementation of energy conservation and carbon reduction projects for key industries such as nonferrous metals, aiming to promote carbon peak in the nonferrous metals industry. However, when we specifically review policy documents related to “green and low-carbon transformation and development of resources and energy,” we find that they fail to adequately recognize the direct support of strategic critical minerals in achieving a green low-carbon energy transition and ensuring resource security.

### 3.2 Policy attention

**Fig. 4:**
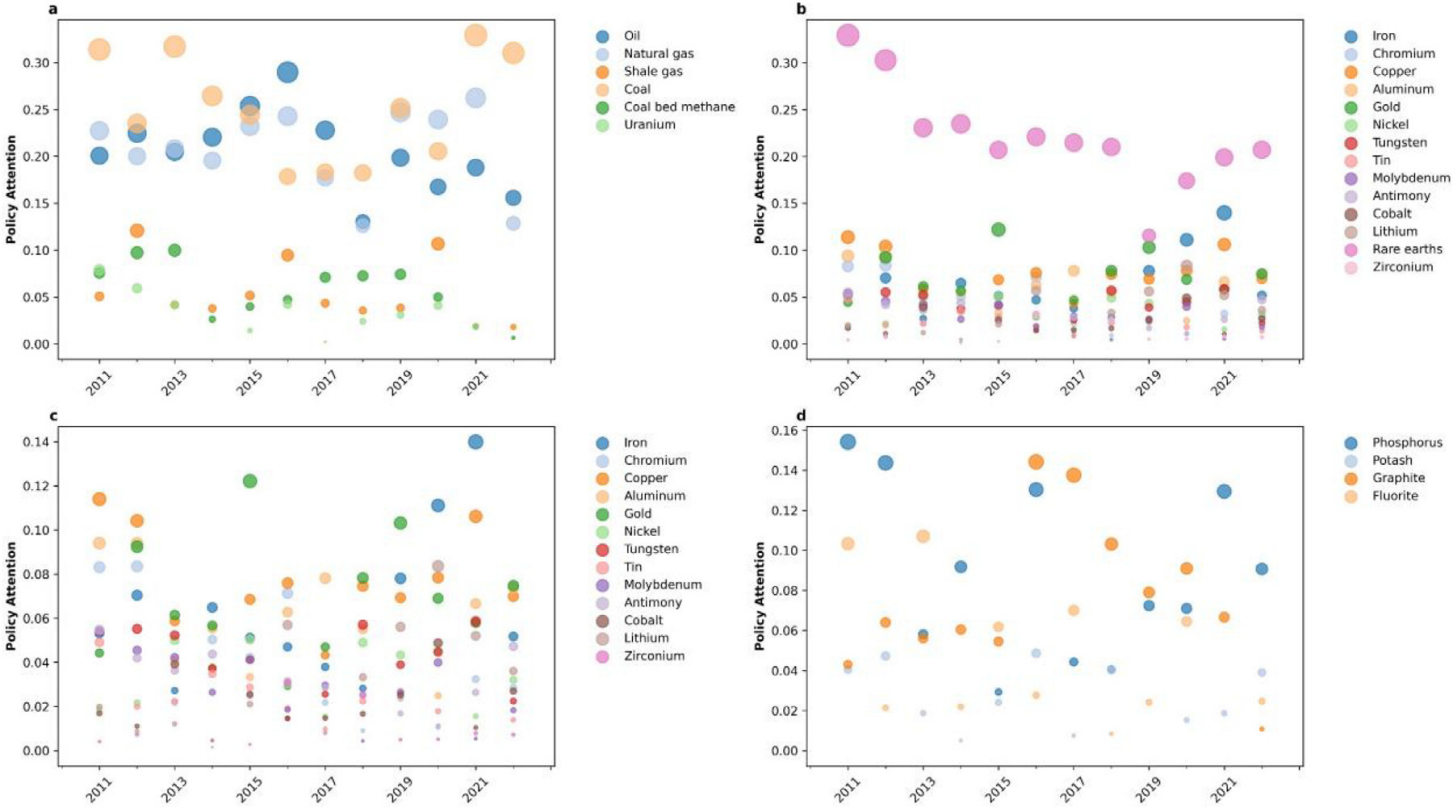
**Policy attention of each type of strategic critical mineral from 2011 to 2022.**The size of each bubble corresponds to the magnitude of policy attention. Different colors represent various types of minerals, as follows: (a) energy policies, (b) metallic policies, (c) metallic policies without rare earths, (d) nonmetallic policies.

To delve deeper into the focus on specific minerals within all strategic critical mineral policies, we employed the TF-IDF algorithm to compute the policy attention scores of individual minerals in each policy text. These scores, ranging between 0 and 1, depict the level of attention attributed to each mineral. Here, the data for 2023 were excluded because the policy text data for 2023 were incomplete. The results are shown in Fig. 4.

Fig. 4a illustrates the policy attention on energy minerals; coal, oil, and natural gas receive a high level of attention ranging from 0.1 to 0.35, while shale gas, coal bed methane, and uranium are given relatively low levels of concern below 0.15. The significant policy attention given to coal, oil, and natural gas highlights their critical importance for China's economic development and national security. This emphasis is driven by China's pursuit of pollution control, environmental protection, and the “dual carbon” goal, leading to the implementation of policies aimed at energy efficiency, emission reduction, and carbon mitigation [45,46].

The policy focus on metallic minerals, as shown in Fig. 4b, is significantly higher for rare earths compared to other metallic minerals, with sustained attention levels above 0.2 over several years. The rare earths play a crucial role as essential raw materials in emerging industries such as high-capacity batteries, displays, and permanent magnets for wind power generation [47]. Simultaneously, due to China's dominant position in rare earth minerals, they have consistently received significant policy attention. In 2011, the State Council issued a policy document “Several Opinions on Promoting the Sustainable and Healthy Development of the Rare Earth Industry”, proposing to adhere to strict controls over total production volumes while optimizing existing stock levels. Additionally, it emphasized expediting the implementation of strategies involving large enterprises and groups, actively promoting technological innovation and establishing a strategic reserve system for rare earths. The implementation of this policy also signifies China's elevation of rare earths to the status of a national strategic reserve. Therefore, within the scope of this study, 2011 represents the peak of policy attention toward rare earth elements. As a result, several related policies were implemented. The robust development of new energy vehicles and wind power generation since 2020 has significantly propelled the surge in demand for rare earth materials [48]. Policy attention on rare earths has risen again. To further analyze the policy attention given to other metallic minerals, we calculated their policy attention excluding rare earths, as shown in Fig. 4c. It is evident that policies place a lesser emphasis on metal minerals other than rare earths, indicating that there are no specific policies that have been implemented for other metal minerals. Notably, zirconium and tin received the least amount of concern, with almost zero attention. The policy formulation has not yet been aligned with the metal minerals listed in the strategic mineral catalog, resulting in a lack of comprehensive attention to metal minerals within the overall policy.

The limited variety of nonmetallic minerals classified as strategic and the lack of comprehensive policies addressing them result in consistently low policy attention, all below 0.16, as depicted in Fig. 4d. Phosphorus and graphite receive relatively higher policy attention. This phenomenon is because phosphate serves as a crucial raw material for fertilizer production, which directly affects China's food security [49]. Additionally, graphite plays a pivotal role as a raw material in developing strategic emerging industries such as batteries and electric vehicles, influencing China's future industrial growth. Consequently, policies place emphasis on these two nonmetallic minerals [50].

### 3.3 Policy strength

**Fig. 5:**
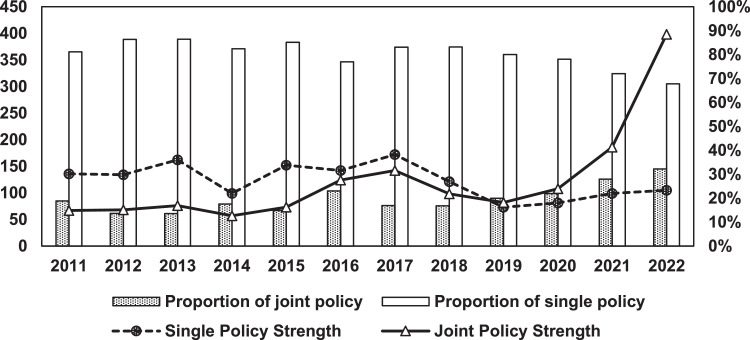
**Policy agency synergy of China's strategic critical minerals policy from 2011 to 2022**.

**Fig. 6:**
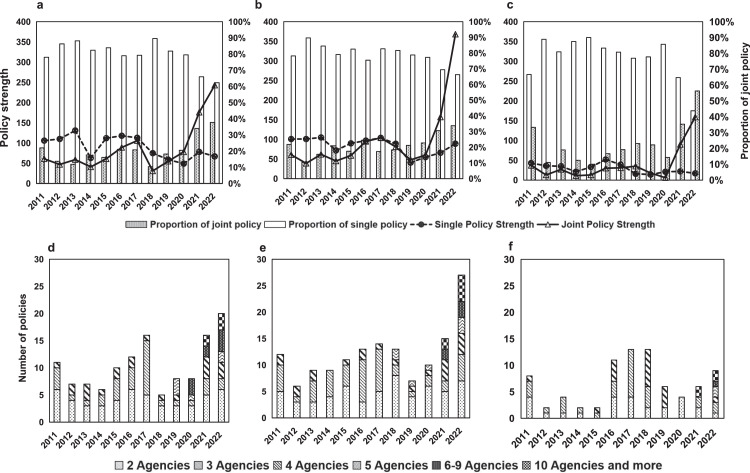
**Policy agency synergy of three types of strategic critical minerals from 2011 to 2022**. Subfigures (a) to (c) represent energy minerals, metallic minerals, and nonmetallic minerals, respectively. Subfigures (d) to (f) represent the distribution of the number of agencies of energy, metallic, and nonmetallic mineral policies, respectively.

We categorized China's strategic critical mineral policies into five types according to the policy strength evaluation indicator system described earlier. We then conducted an analysis of the evolution of single-sector policy versus joint policy and calculated the sum of policy strengths for years corresponding to each of the two scenarios in order to explore the synergy between policy agencies over time. The outcomes are presented in Fig. 5, Fig. 6.

In general, there has been an increase in the proportion of policies jointly issued for strategic critical minerals, indicating progress toward a more cross-sector approach to development. Although the proportion of single-sectoral policy consistently exceeds that of joint policy, a shift occurred after 2019, when the joint policy strength surpassed that of single policy strength. This phenomenon indicates that in the early period, the government believed that leveraging the expertise of specific agencies would effectively address challenges, thereby resulting in a high single-policy strength. However, as the complexity surrounding strategic critical mineral issues grew, it became evident that a single-sectoral policy formulation could no longer adequately address these challenges. Consequently, the Chinese government gradually started cross-sectoral collaboration to develop more authoritative policies for guiding strategic critical mineral development.

Fig. 6 shows a fluctuating increase in the proportion of joint policies for three types of mineral policies, which can be explained by the improvement in multisectoral cooperation in the decision-making of the three types of mineral policies, reflecting the strengthening of the Chinese government's policy deployment for strategic critical minerals. In this sense, the proposed policy strength index can effectively measure and capture the authority and complexity of policies.

In terms of policy strength, Fig. 6b shows that the joint policy strength of metallic mineral policies grows most significantly compared to the other two types of policies, especially in 2022. We further focused on joint policy issuing and explored changes in synergy between policy agencies by counting the number of issuing agencies. The distribution of the number of agencies of energy, metallic, and nonmetallic mineral policies is shown in subfigures Fig. 6d-f. The majority of policy documents are issued by five or fewer agencies. Small-scale policy synergy was characteristic of the strategic critical minerals policy formulation process. This is due to the complexity of the process of policy synergy, which requires trust and rich information exchange among agencies to help resolve conflicts in the process of policy synergy, so there are only a few agencies involved in the early stages [51]. With the increasing number of stakeholders involved in strategic critical mineral policies, more agencies are involved in the policymaking process to balance the interests of various policy entities. In the case of metallic mineral policies, for instance, the involvement of more than five agencies in policymaking emerges after 2021. In particular, the number of policy documents jointly issued by ten or more agencies reached five in 2022, which is the main reason their policy strength grew significantly more than the other two types of mineral policies in 2022.

### 3.4 Policy synergy network

**Fig. 7:**
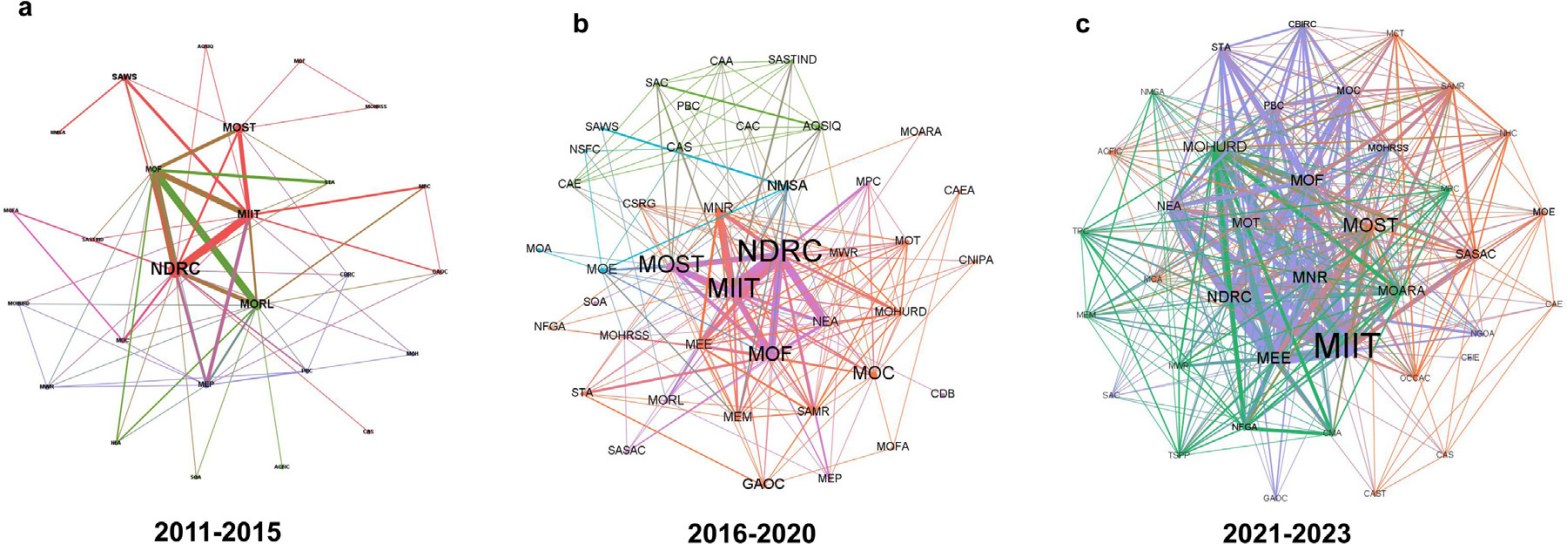
**Synergy network for strategic critical minerals policy agency from 2011 to 2023 in each period**.

We analyzed the collaboration and transformation of government agencies during the development of strategic policies on critical minerals in China, focusing on the evolution of interagency synergy. The network of policy synergy relationships at each stage is visualized in Fig. 7, and network characteristics such as density and average clustering coefficient were quantified and are presented in Table 4, with the department abbreviations listed in Table A1. A staged analysis of the policy synergy network is also provided, and Table A2 details the centrality of nodes within the network, highlighting the key agencies involved in different periods. In Fig. 7, each node represents a government agency that participated in the joint issuance of the strategic minerals policies for the respective period. The node sizes are based on betweenness centrality, with larger nodes indicating a greater role in coordinating policymaking among agencies. It is noteworthy that China has undergone governmental departmental reforms, including mergers, splits, and the renaming of various departments. These changes reflect a shift in the functions and objectives of government departments. To conduct a more in-depth analysis of policy evolution, we refrain from integrating the names of former and current departments within this study.

**Table 4:** **Network characteristics of synergistic relationships in agencies for strategic critical minerals policy across phases from 2011 to 2023**.

Period	Density	Average Clustering Coefficient	Average Path Length	Number of Communities	Nodes
2011–2015	0.218	0.756	1.991	4	26
2016–2020	0.237	0.785	1.923	4	41
2021–2023	0.546	0.848	1.454	3	38

The work on strategic critical minerals is characterized by its complexity and cross-domain nature. As shown in Table 4, the density of the policy agency synergy network continues to increase with the evolution of stages, while the average clustering coefficient also continues to slightly grow. This indicates that nodes are becoming more aggregated and that agencies present a closer cooperative relationship. At the same time, the gradual reduction of average path length reflects the improvement of communication efficiency among government agencies.

The policy synergy network of minerals policies in 2011–2015 is depicted in Fig. 7a, in which the National Development and Reform Commission (NDRC) prominently occupies a central position, signifying its pivotal role in formulating strategic critical minerals policies. The NDRC holds a central position in the network as it formulates and coordinates the implementation of national economic and social development strategies, medium- to long-term plans, and annual plans. In addition to the NDRC, the Ministry of Land and Resources (MLR), the Ministry of Industry and Information Technology (MIIT), and the Ministry of Science and Technology (MOST) are also leading agencies in the network. The MLR is responsible for the protection and rational utilization of natural resources, including land and mineral resources, while the Department of Raw Materials in the MIIT manages the industrial aspects of raw materials such as iron and steel, nonferrous metals, rare earths, etc. The MOST is responsible for formulating and implementing policies related to science and technology innovation in the country, signifying China's support for the advancement of the mineral industry through a multitude of science and technology innovation measures in this period.

As Fig. 7b shows, the number of nodes participating in the network increased from 2016 to 2020, as new agencies joined the policy issuing process, resulting in closer relationships among existing agencies. This development can be attributed to China's departmental reforms in 2018, which resulted in the establishment of new ministries such as the Ministry of Natural Resources (MNR), the Ministry of Ecology and Environment (MEE), and the Ministry of Emergency Management (MEM). Consequently, these newly established ministries were integrated into the policy synergy network to facilitate synergistic development among agencies. Additionally, the dominant sector of the network has undergone a slight shift, with the Ministry of Finance (MOF) and the Ministry of Commerce (MOC) emerging as key players alongside the NDRC, MIIT, and MOST. Though not responsible for the release of the largest number of policies, the MOF and the MOC are increasingly involved in joint policy formulation, indicating China's utilization of a wider range of fiscal tools as well as trade investment and market mechanisms to support and manage mineral development. Furthermore, the interconnections between the MOF, MIIT, NDRC, and MOST are more pronounced. This connection indicates a higher frequency of joint policy releases among these departments and the gradual establishment of policy synergy in such areas as finance and taxation, industrial development, investment promotion, and scientific and technological innovation.

Between 2021 and 2023, the policy synergy network became closer, and the number of lead agencies increased significantly, further enhancing synergies between policy sectors while advancing the development of the policy system. As shown in Fig. 7c, China's focus on the development of strategic critical minerals in the industrial and industry sectors is evident, as the MIIT holds a central position in the network. In collaboration with several other ministries, the MIIT has formulated various specialized development plans pertaining to the strategic critical minerals industry during the 14th Five-Year Plan period. Table A2 shows that the MOST has risen in the ranking of centrality, ranking second. The MEE has exhibited a significant rise in its ranking of centrality, emerging as the third most central agency within the network. This change implies China's greater emphasis on promoting synergistic development between mining and ecology during the 14th Five-Year Plan period. Meanwhile, we find that the State-owned Assets Supervision and Administration Commission of the State Council (SASAC) becomes a more central node in the network. During the 14th Five-Year Plan period, China has continued to promote the restructuring of central enterprises, focusing on integrating specialization and industrialization. In line with this objective, the China Rare Earth Group was formed on December 23, 2021, while the establishment of the China Mineral Resources Group Corporation took place on July 25, 2022. These strategic initiatives aim to safeguard the critical position of rare earths and other strategic mineral resources, facilitating their sustainable development and reshaping China's industrial pattern for strategic minerals. During the 14th Five-Year Plan period, strategic critical mineral policymaking places an emphasis on industry and industrial development, while leveraging the power of central enterprises to enhance China's mineral industry competitiveness.

**Fig. 8:**
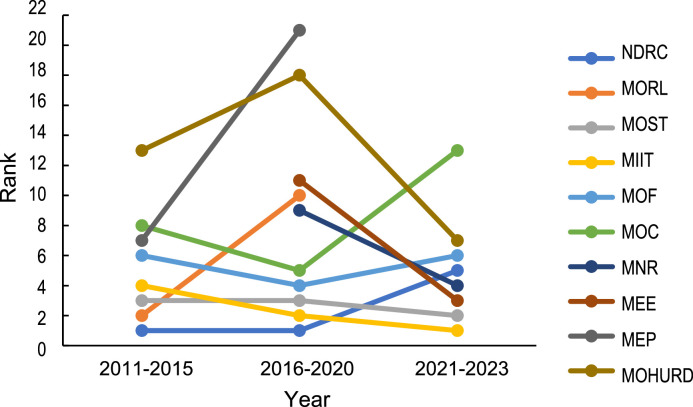
**Top 10 agencies of betweenness centrality in the synergy network**.

We also identified the key agencies that played central roles in the synergy network during each period. Fig. 8 presents the top 10 agencies based on their betweenness centrality in each of the three periods. The MIIT, NDRC, and MOST have consistently been the core agencies within the policy synergy network, indicating their pivotal role in shaping China's strategic critical minerals policy. The centrality of the NDRC has diminished in terms of sectoral changes, while those of the MIIT has increased, indicating a shift in policymaking of strategic critical minerals toward greater emphasis on the industrial and manufacturing aspects. Following the 2018 departmental reform of the State Council, the MORL was abolished and replaced by the MNR, which has since taken its place within the synergy network. Through continuous synergistic development among various agencies, a policy formulation system for strategic critical minerals in China has been established during the 14th Five-Year Plan period, which forms a network of policy agencies centered on the MIIT, MOST, MEE, MNR, NDRC, and MOF.

### 3.5 Discussion

Enterprises and technology have always been the focal points of China's strategic critical minerals policy deployment, with green and low-carbon initiatives becoming the new focus of attention in the country's strategic critical minerals policies since the 14th Five-Year Plan period. This observation aligns with the Chinese government's recent efforts in addressing challenges posed by climate change and prominent issues related to resource and environmental constraints. The Chinese government has set ambitious goals, such as the “dual carbon goals” [52] and implemented a series of policy measures to tackle these challenges. The 19th National Congress of the Communist Party of China proposed to “advance the energy production and consumption revolution, and construct a clean, low-carbon, secure, and efficient energy system” [53]. Concurrently, it prioritized the strategic development of new energy and new energy vehicles as emerging industries, formulating a series of strategic objectives.

Policies place a strong emphasis on energy minerals and rare earths. Despite China's policy shift toward green and low-carbon development, the analysis of the policy attention given to 24 strategic critical minerals reveals that the government's focus remains highest on coal, oil, and natural gas, the three traditional energy minerals. Meanwhile, there appears to be insufficient policy attention directed toward metallic and nonmetallic minerals. Moreover, the policy content primarily centers on energy conservation and carbon reduction objectives for the mineral industry, which exhibits high levels of energy consumption and carbon emissions. Conversely, limited attention is given to the strategic significance and security assurance of strategic critical minerals under the backdrop of the “dual carbon goals.” This misalignment between policy content and policy direction poses a challenge in the context of the current green-oriented transition of energy and its impact on resource security. Additionally, in the top 20 keywords in the co-occurrence networks, keywords such as “materials” and “equipment”, which represent the midstream and downstream industries, emerged as significant nodes during the period from 2016 to 2020. However, these keywords did not rank in the top 20 keywords after 2020. This observation implies that there remains space for strengthening the policy's emphasis on the middle and downstream segments of critical mineral supply chains. Addressing this challenge necessitates the development of a comprehensive plan for strategic critical minerals in China, fostering a bidirectional synergy between policy direction and content to ensure a stable supply.

The synergy among China's strategic critical minerals policy sectors has been continuously strengthening, and the policy formulation system is gradually improving. Through the integration and establishment of new government departmental organizations, China has forged a synergistic network of policy sectors, with the MIIT, MOST, MEE, MNR, NDRC, and MOC serving as the core departments. By enhancing collaboration among existing national governmental departments, China has established a coordinated approach to policies encompassing industrial development, technological innovation, environmental protection, natural resources, investment, and finance, as well as import and export, with the aim of guiding and managing the development of strategic critical minerals.

**Fig. 9:**
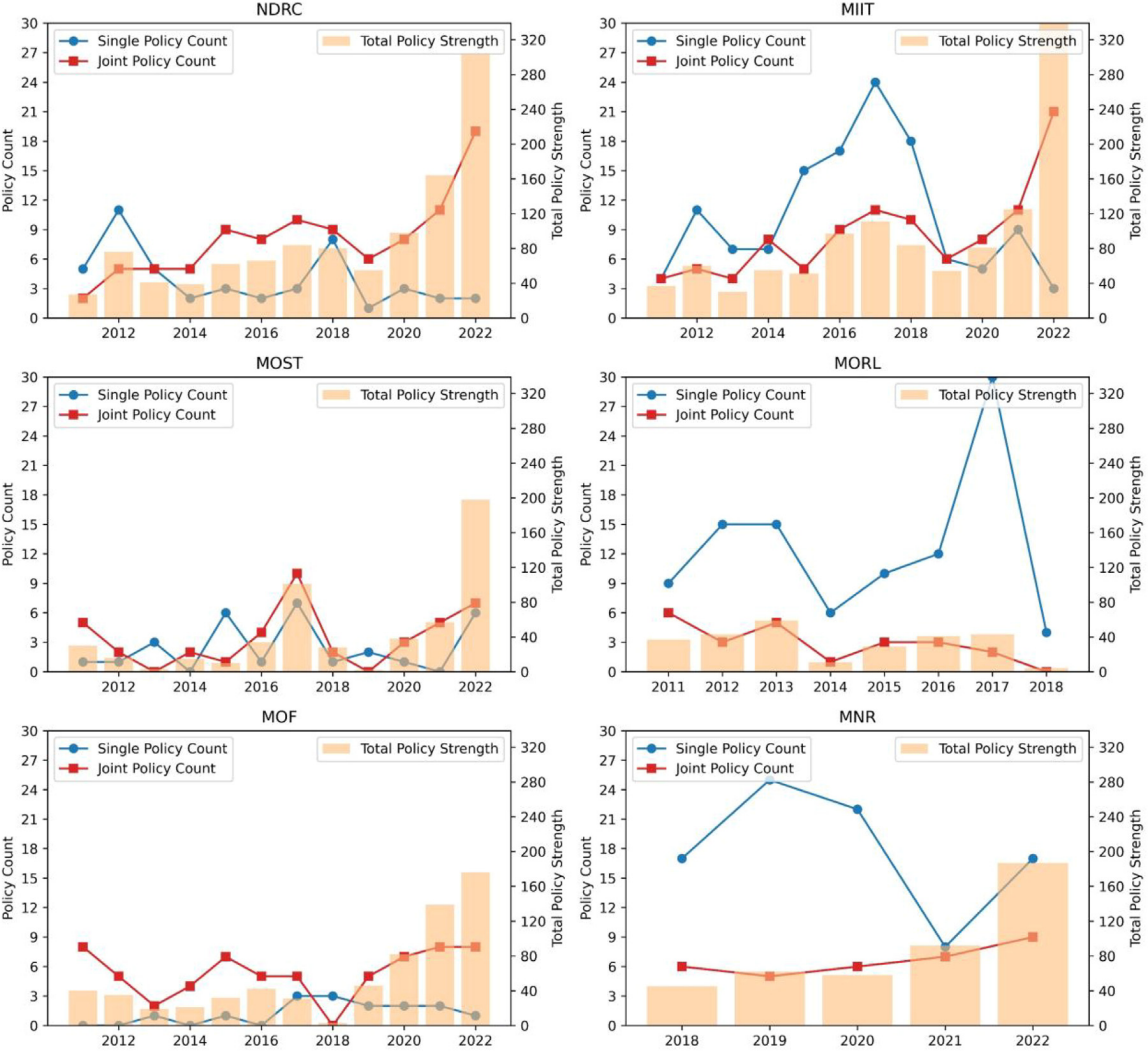
**Comparative analysis of policies issued by the Ministry of Natural Resources vs. other government agencies**.

The MNR, which is currently responsible for policy deployment related to natural resources, does not occupy the most central position within the synergistic network of policy sectors. Firstly, as shown in Fig. 9, by comparing single policies with joint policies issued by the MNR, it can be seen that the number of jointly issued policies is significantly lower than the number of policies issued by a single department, though it is also showing an upward trend. Secondly, the overall policy effectiveness of the MNR still falls behind that of pivotal departments, which assume an intermediary role within the policy network. This indicates that although the MNR has made gradual progress toward fostering cross-sectoral synergy in the policy development process, its collaborative policy development with other departments remains insufficient in terms of its policy strength. Consequently, the MNR has not fully assumed its central role as the primary department responsible for resource management within the policy framework.

## 4 Conclusions

The significance of strategic critical minerals in the global transition toward green and low-carbon energy has attracted considerable attention from many countries. As a result, it is crucial to analyze the evolution of strategic critical minerals policy systems to identify key policy themes and deficiencies within the policymaking framework. However, existing literature on China's strategic critical minerals lacks a systematic and comprehensive examination of the policy system's evolution. To fill this gap, this study employs text mining and social network analysis methods to process a large volume of policy text data and explore policy evolution through the lens of “policy theme-policy synergy.” Our key findings are as follows:

(1) Enterprises and technology have consistently remained at the forefront of China's policy agenda, with a new emphasis on green and low-carbon initiatives considering the carbon peaking and carbon neutrality goals. The government should incorporate technological innovation, comprehensive industry chain management, and other strategies into policy formulation to further enhance the pivotal role of enterprises and technology in driving the energy transition process, thereby facilitating low-carbon transformation and development of the mineral sector through advancements in industrial technology.

(2) The significant policy attention given to rare earth minerals reflects China's comprehensive policy framework and extensive management experiences in the development and governance of the rare earths industry. In order to ensure resource security while achieving carbon neutrality, China can leverage its extensive management expertise in the rare earths sector and implement policy reserves for other minerals. China should enhance the significance of strategic critical minerals in policy formulation and ensure resource supply security through measures such as protective mining and import and export management, among others.

(3) The number of ministries involved in strategic and critical minerals policies has increased, with more jointly issued policies and greater intensity of policy issuance, resulting in a tighter policy synergy network. The involvement of multiple government agencies in collaborative policy development has emerged as a prevailing practice, enhancing interagency communication efficacy. Gradually, a policy synergy network has been established, with the Ministry of Industry and Information Technology, Ministry of Science and Technology, Ministry of Ecology and Environment, Ministry of Natural Resources, National Development and Reform Commission, and Ministry of Commerce serving as core departments. Through policies related to industrial development, scientific innovation, environmental resources management, and finance and taxation, among others, these efforts aim to effectively guide the development of strategic critical minerals. Simultaneously, given the extensive scope of strategic key minerals and their long industrial chain, future policy formulation should continue to assume a multisectoral coordination role in order to effectively support the holistic development of the entire industrial chain.

(4) The Ministry of Natural Resources has yet to demonstrate its central position as the leading department responsible for managing the development of all types of resources. It is imperative to enhance the pivotal role of the Ministry of Natural Resources as a key department by actively establishing a comprehensive policy framework for strategic critical mineral policies, prioritizing cross-level and cross-sectoral coordination, and enhancing policy feasibility.

## Declaration of competing interest

The authors declare that they have no conflicts of interest in this work.

## Figures and Tables

**Table:** **Table A1Name of department abbreviations**.

Name of department	Abbreviations	Name of department	Abbreviations
Ministry of Land and Resources	MORL	The State Administration of Science, Technology and Industry for National Defense	SASTIND
State Administration of Work Safety	SAWS	Ministry of Natural Resources	MNR
Ministry of Industry and Information Technology	MIIT	Ministry of Finance	MOF
National Development and Reform Commission	NDRC	Ministry of Transport	MOT
Ministry of Environmental Protection	MEP	China Banking and Insurance Regulatory Commission	CBIRC
Ministry of Human Resources and Social Security	MOHRSS	Chinese Academy of Science	CAS
National Mine Safety Administration	NMSA	Chinese Academy of Engineering	CAE
State Oceanic Administration	SOA	Ministry of Commerce	MOC
Ministry of Science and Technology	MOST	Ministry of Education	MOE
Ministry of Housing and Urban-Rural Development	MOHURD	National Audit Office	NAO
Administration of Quality Supervision, Inspection and Quarantine	AQSIQ	General Administration of Customs	GAOC
State-owned Assets Supervision and Administration Commission of the State Council	SASAC	State Council	SC
China Earthquake Administration	CEA	The National People's Congress	NPC
China Association for Science and Technology	CAST	Tariff Committee of the State Council	TCOSC
National Forestry and Grassland Administration	NFGA	State Taxation Administration	STA
National Energy Administration	NEA	Ministry of Foreign Affairs	MOFA
Office of the Central Cyberspace Affairs Commission	OCCAC	Ministry of Agriculture	MOA
National Health Commission	NHC	All-China Federation of Industry and Commerce	ACFIC
Ministry of Culture and Tourism	MCT	China Banking Regulatory Commission	CBRC
China Meteorological Administration	CMA	The Safety Production Committee	SPC
Ministry of Agriculture and Rural Affairs	MOARA	Ministry of Water Resources	MWR
State Administration for Market Regulation	SAMR	China Food and Drug Administration	CFDA
Ministry of Public Security	MPC	National Mine Safety Administration	NMSA
China State Railway Group Co., Ltd	CSRG	Ministry of Emergency Management	MEM
Ministry of Ecology and Environment	MEE	People's Bank of China	PBC
China Atomic Energy Authority	CAEA	National Food and Strategic Reserves Administration	NFSRA
National Natural Science Foundation of China	NSFC	China Federation of Industrial Economics	CFIE
Certification and Accreditation Administration	CAA	The Supreme People's Court	TPC
Standardization Administration of China	SAC	National Government Offices Administration	NGOA
China Development Bank	CDB	Ministry of Civil Affairs	MCA
China Securities Regulatory Commission	CSRC	National Bureau of Statistics	NBS
China National Intellectual Property Administration	CNIPA	The Supreme People's Procuratorate	TSPP
Ministry of Health	MOH		

**Table:** **Table A2Three period of Betweenness centrality in policy agency synergy networks**.

2011–2015	Betweenness Centrality	2016–2020	Betweenness Centrality	2021–2023	Betweenness Centrality
NDRC	0.256	NDRC	0.190	MIIT	0.120
MORL	0.216	MIIT	0.150	MOST	0.076
MOST	0.208	MOST	0.113	MEE	0.059
MIIT	0.131	MOF	0.093	MNR	0.050
SAWS	0.093	MOC	0.081	NDRC	0.044
MOF	0.082	GAOC	0.076	MOF	0.041
MEP	0.071	NMSA	0.063	MOHURD	0.041
MOC	0.062	MOE	0.056	MOT	0.037
MWR	0.060	MNR	0.039	MOARA	0.029
CBRC	0.059	MORL	0.037	SASAC	0.026
GAOC	0.058	MEE	0.036	NEA	0.025
PBC	0.049	MEM	0.035	PBC	0.025
MOHURD	0.018	NEA	0.035	MOC	0.023
NEA	0.016	AQSIQ	0.034	MOE	0.022
STA	0.014	CAS	0.031	STA	0.019
SASTIND	0.013	MWR	0.027	MOHRSS	0.019
MOH	0.005	MOT	0.019	CBIRC	0.014
MPC	0.004	MOHURD	0.019	NFGA	0.013
AQSIQ	0.003	SAMR	0.017	SAMR	0.011
SOA	0	MPC	0.017	CMA	0.011
MOFA	0	MEP	0.017	OCCAC	0.011
CAS	0	STA	0.016	MCT	0.011
ACFIC	0	CAC	0.014	NHC	0.010
MOHRSS	0	MOHRSS	0.011	MEM	0.010
MOE	0	SAWS	0.011	MWR	0.010
NMSA	0	CNIPA	0.011	TPC	0.008
		CAEA	0.008	TSPP	0.006
		MOFA	0.006	MPC	0.005
		SOA	0.005	NGOA	0.005
		SASTIND	0.003	SAC	0.005
		CAE	0.002	CFIE	0.005
		CAA	0.002	MCA	0.004
		SAC	0.001	ACFIC	0.004
		NSFC	0.001	NMSA	0.004
		MOA	0	CAS	0.004
		SASAC	0	CAE	0.004
		CSRG	0	CAST	0.003
		CDB	0	GAOC	0
		PBC	0		
		MOARA	0		
		NFGA	0		
